# Artificial Intelligence and brain

**DOI:** 10.6026/97320630014038

**Published:** 2018-01-31

**Authors:** Paul Shapshak

**Affiliations:** 1 Division of Infectious Diseases and International Health, Department of Internal Medicine, University of South Florida, Morsani College of Medicine, Tampa, FL 33606, USA;

**Keywords:** Artificial Intelligence (AI), neuroscience, biomedicine, ecosystem, brain, cognitive architecture, self-motivated behavior, symbol grounding, language grounding, cognitive science, games, event schemata, deep learning, mirror neuron, shadow neuron, neuron manifold, neuron group, neuron colony, gene expression, research, development, topology, manifold, fiber bundle, computer, machine, cognition, awareness, consciousness, quantum computers, robotics, co-robotics (cobots), Godel, paradigm shift, hall of mirror neurons, neuropsychiatric disease

## Abstract

From the start, Kurt Godel observed that computer and brain paradigms were considered on a par by researchers and that researchers had misunderstood his theorems. He hailed with displeasure that the brain transcends computers. In this brief article, we point out that Artificial Intelligence (AI) comprises multitudes of human-made methodologies, systems, and languages, and implemented with computer technology. These advances enhance development in the electron and quantum realms. In the biological realm, animal neurons function, also utilizing electron flow, and are products of evolution. Mirror neurons are an important paradigm in neuroscience research. Moreover, the paradigm shift proposed here - 'hall of mirror neurons' - is a potentially further productive research tactic. These concepts further expand AI and brain research.

## Background

AI, robotics, co-robotics (cobots), and quantum computers were briefly reviewed previously, and their achievable utilities were advocated for virology, community health, clinics, research, and development [1]. Antecedent theories as well as key concepts from individuals who set the stage for AI, robotics, and quantum computers were recognized, including Vannevar Bush, Alan Turing, John McCarthy, Norbert Wiener, John von Neumann, John Nash, Kurt Godel, and Richard Feynman, to name a few. It is of further interest to briefly mention additional steps taken to advance AI vis a vis brain.

## Artificial Intelligence

AI generally is subsumed by approaches including neural networks, machine learning, deep learning, etc. There can be more than 100 layers in these learning algorithms. The layering organization is often input, hidden, and output architectures [2, 3, 4, 5]. There are many computer programs and architectures that exemplify uses of AI. Some computer programs are capable of learning to play computer games, which is an important development for AI and potentially, in time, working towards awareness. For example, work is being done to unify a theory of cognition involving sub-symbolic computation. Schrodt and colleagues [6] presented Semlincs, architecture with an instrument that controls cognition. This agent acquires assemblies with rules garnered from its own independent continual sensorimotor experiences. The essential ingredient is that this instrument attains knowledge of interactions with its ecosystem to design and regulate sub-environments within that ecology. This is accomplished in a self-driven manner that is adaptable, goalguided, and self-motivated. It should be noted that this is a step forward, different from other preceding cognitive and symbolic architectures.

The persona of Mario, derived from a clone of the well-known children's computer game, 'Super Mario Brothers', is developed using Semlincs. This demonstrates the program's versatile selfgoverning and knowledge-gathering control of behavior. There is a trajectory of improvement of production rules, resulting directly from sensorimotor experiences. This exemplifies a key component in the development of AI as well as towards cognitive computational theory and practice [6, 7, 8].

## Brain and Mirror neurons:

Frequently, AI is fueled by the study and simulation of neuronal function specifically, and promoted by neuroscience, in general. There is reciprocity, and brain research progress is also inspired by developments in AI. A heated topic and jump forward in neuroscience during the last few decades, is the study of mirror neurons in the primate brain and this is a highly productive component of brain research [9, 10, 11, 12]. A PubMed search for, 'mirror neuron system' produced 1,139 publications since 1967 and searches for, 'cortical mirror motor neurons' resulted in 365 publications since 1987 [13]. In addition, possibly, several publications prior to the specific use of the terminology, mirror neurons, were unrecognized in the early publications, in differing contexts, in such searches.

Mirror neurons occur in many brain regions and affect, control, and mirror particular sensi-motor activities across a wide range of interconnections. Several brain areas with mirror neurons include dorsal premotor and primary motor cortex, rostral division of the ventral premotor cortex (area F5), and inferior lateral and ventral intraparietal areas of the parietal lobe, among others. Mirror neurons are apparently involved or activated when anyone (monkey or human, for example) performs specific motor actions as well as when anyone witnesses another individual executing comparable or equivalent actions. Auditory as well as visual input can both activate mirror neuron clusters [9, 14]. It should be noted that mirror neurons are anticipated to carry out functions that are different from cybernetic feedbackcontrol neurons, which were originally postulated by Norbert Wiener [15]. Remote from any amorphous functions, mirror neurons are involved in inception, recollection, learning, and repeat of actions (whether voluntary, visualized, or heard) whereas distinctly other neurons are involved in feedbackcontrol. It may be surmised that volitional remembered motor actions, would be anticipated to involve such mirror neurons as well.

Since there are many mirror neuron foci, as mentioned further in this article, the modes of neuron development, post-birth as well as in adults are of relevance [9]. Neurons can be produced from stem cells. This differentiation involves in situ processes and also achievable step of migrating stem cells to other brain regions, where they can differentiate, based on several types of signaling inputs. Moreover, the library of DNA expression information during fetal development and in the adult is germane for successful differentiation of mirror neurons [16, 17]. In the adult, stem cells are well known to exist in the rodent and human dentate gyrus of the hippocampus, subventricular zone of the lateral ventricles, and olfactory bulb [17, 18].

Possibly, while still in semi-differentiated states, some nascent neuron groups may migrate further away from one region to another region, spinning out their axonal threads along the way. When their migratory patterns are finally accomplished, they could complete differentiation into the precise neuron-types needed - in this example, specifically, mirror neurons. Thus, mirror neurons, upon completing their differentiation processes, could come to reside at some distance from the original particular motor neuron groups and maintain their long-range specific communication links with their respective motor neuron assemblies via their spun axons. Some mirror neuron activities could include what might be termed, shadow neurons, which could function as topological manifolds overlapping or layering in proximity to other neurons, for example, as sheets of tangential or enveloping neuron clouds. In addition, various mirror neurons may be components of ensemble, satellite, cluster, swarm, or colony groups. Moreover, some mirror neurons may be directly linked, single motor neuron to single mirror neuron (besides any other sensory-related neuronal circuits within which they also may function), thus individually, isomorphically linked with each neuron that they mirror. In the latter case, mirror neurons may indeed function individually, with greater neurobiological precision and exactitude than heretofore surmised. The precise number or extent of interactions of motor with mirror neurons remains an ongoing challenge towards research in brain function.

## Brain disease and mirror neurons

In support of the physiological importance of mirror neurons, is their possible involvement in human brain diseases, for example, in amyotrophic lateral sclerosis (ALS). Eisen and colleagues, in 2015, proposed that ALS and fronto-temporal dementia might be associated with particular mirror neuron dysfunction. In ALS, possibly, empathy, language, gesture, and imagery may be affected by mirror neuron error [19].

Mirror neurons appear to be involved in additional brain diseases and a few examples follow. Alzheimer's disease is hypothesized to have a link with motor function and this hypothesis is termed the Embodied Cognition Hypothesis. This hypothesis proposes that perception representations are coupled with actions. Mirror neurons may weigh heavily in this regard. Recently, mirror neuron integrity was studied in several categories of aging people: Alzheimer disease, Mild Cognitive impairment with hippocampal atrophy, and normal aging. In Alzheimer's disease, mirror neurons are explicitly damaged. In Mild Cognitive Impairment, preserved cognition might be due to the safeguarding of anterior mirror neurons. This may complement posterior mirror neurons during their initial decline in Mild Cognitive Impairment. In normal aging, mirror neurons are generally conserved [20]. Using fMRI studies in autism and normal individuals, when participants match brain activity of other individuals; mirror neurons are identified as responsible for emotions, empathy, intention recognition, and complex cognition [21]. Mirror neuron malfunction is also implicated in schizophrenia, which is apt, due to the extensive psyche dysfunction in this complex of disease types and subtypes [22].

## Hall of mirror neurons and Paradigm shift:

Based on mirror neuron function in brain, a paradigm shift in relation to consciousness and cognition is proposed that could be termed the 'hall of mirror neuron' paradigm. If one postulates that there may be several mirror neuron foci of other mirror neuron foci, and that this could occur at several levels, in a chainlike manner, then such arrays could evolve further away from the initial motor-sensory actions that initiated the complementarity of motor vs. mirror neuron groups. In this way, the more distant such mirror neuron foci become increasingly separated from the initial motor-sensory complexes or groups. Such processes could contribute to cognition and consciousness. This could be a mode for evolution or development of more 'theoretical' cognition, (more theoretical in that the more distant mirror neuron foci are no longer directly tied to immediate sensory-motor input neurons). By the same token, this paradigm shift could be applied to AI construction and inventiveness. The geometric expansion of such 'hall of mirror neuron' group interactions (with the inclusion of exceedingly many axonal and synaptic interactions) may soon exhaust the capacity of classical computers, supporting a further need for quantum computer development.

## Topological model for hall of mirror neurons:

Research on brain is a natural setting for application of many different mathematical methods. Thus, concepts in differential topology of manifolds [23, 24, 25, 26] may be of use for neuronal interactions, arrangements, and clusters - viz. mirror neurons. Interconnections between motor neuron foci and their mirror neurons may be considered as maps between manifolds. These interconnections may be quantified by intensities of interactions in each direction. The additional interactions of the motor and sensory neuron foci with each mirror neuron group may be considered analogous to fiber bundles. The quantitative intensities of these fiber bundles may be based also on intensities of their e.g. sensory input interactions. If the 'hall of mirror neuron' paradigm is useful, then this type of algebraic and geometric topology will have an additional role in the quantitative analysis of mirror neuron topological interactions. A corollary to these deliberations is that there may be partial overlaps among various mirror neuron foci in the 'hall of mirror neuron' paradigm. Should this be the case, and then clearly, the topological characterization will resemble the central issues of mathematical mapping in the elucidation of fiber bundles. Moreover, since fiber bundles themselves may function as manifolds, the additional level of mirror neuron foci may be further treated as fiber bundles, and so on. In this way, topological methods assist in modeling such brain activity including terms such as J topology, M manifold, B base space mirror neuron cluster, ∏ mapping, ∏-1 inverse mapping, arrow mapping à.

Consequently,

Mi ←πij-1à→ij Bj(Mi) ←πjk-1→πjk Bk(Bj(Mi)) ←πkl-1→πkl Bl(Bk(Bj(Mi))) etc…

In addition to interaction in one dimension, it should be noted that interactions among various mirror neurons in the hall of mirror neuron paradigm, could occur multiply, in two or three dimensions, in planar or in cubical arrays, respectively. This further increases the complexity of this model to perhaps contribute to brain and AI modeling.

## Artificial Intelligence-brain heuristic comparison

These approaches will also be productive for use in AI progress and development. The possibility of producing AI-type mirror foci is an additional challenge and the mirror neuron concept is being applied to AI to further the sophistication and subtlety of AI [27, 9]. The hall of mirror neuron paradigm could implement topological groups or swarms of architectures of foci based on these concepts and further widened.

Kurt Godel, perhaps the greatest mathematician since Euclid and Archimedes, published his completeness theorems and analysis of the consistency hypothesis of the continuum in the early 20th century [29, 30]. This work was conceptually in contrast to studies by David Hilbert and others that claimed mathematical logic could prove the completeness of mathematics. Later, Paul J. Cohen incontrovertibly confirmed Godel's work during subsequent decades [31, 32]. Many researchers in AI as well as in neurosciences had assumed basically that AI and brain function could be described using axiomatic algorithms. It had been presumed that there could be no calculation that was inimitable to this standard approach. Moreover, AI and brain have been considered comparable in that it was presumed implicit that both could be reduced to direct axiomatized algorithms and paradigms.

However, Kurt Godel assailed these standpoints after publication of his theorems in 1931. Godel perceived that mathematicians, computer experts, and brain researchers misunderstood his work [33]. On the one hand, mathematicians and computer experts were not acknowledging the inability of computers to resolve what are termed unsolvable propositions. On the other hand, Godel cautioned brain researchers that the human brain was not a computer and was more complex than computers. Godel himself promulgated the notion that the brain transcends computers, i.e. that the brain is more than a set of reductionist computer algorithms.

## Conclusions

Research investigations on AI (computer programs) and brain organization (biological neuronal organization and function) are helpful to each other, where ideas and architectural constructs from one, stimulate work in the other. These endeavors comprise multiple assemblies of methodology, language, architecture, and technology with the fundamental problem of attempting to define and understand cognition and consciousness. The use of the paradigm shift of 'hall of mirror neurons' may help bridge the gap between brain and AI. Computer professionals and brain researchers need to address the limitations of computers and need to comprehend the exceeding complexity of the brain and consciousness. Paradigms require developments that conceptualize beyond current computer models. Quantum computers need to be developed that are capable of handling the complexities of such tasks.

## Conflict of Interest

The author reports no conflicts of interest.
